# Survival of AIDS Patients Treated with Traditional Chinese Medicine in Rural Central China: A Retrospective Cohort Study, 2004–2012

**DOI:** 10.1155/2015/282819

**Published:** 2015-03-02

**Authors:** Yantao Jin, Xin Wang, Zhengwei Li, Ziqiang Jiang, Huijun Guo, Zhibin Liu, Liran Xu

**Affiliations:** ^1^Department of AIDS Treatment and Research Center, The First Affiliated Hospital of Henan University of Traditional Chinese Medicine, Zhengzhou 45000, China; ^2^National Center for AIDS/STD Control and Prevention, Chinese Center for Disease Control and Prevention, Beijing 102206, China; ^3^School of International Education, Zhengzhou Railway Vocational and Technical College, Zhengzhou 450000, China; ^4^Key Laboratory of Viral Diseases Prevention and Treatment of Traditional Chinese Medicine of Henan Province, Zhengzhou 450000, China

## Abstract

This study aimed to explore the survival of AIDS patients treated with traditional Chinese medicine (TCM) in addition to combined antiretroviral therapy (cART) and of AIDS patients treated with cART. Data of patients taking cART between 30 October 2003 and 30 October 2004 in the National TCM HIV Treatment Trial Program area were retrospectively analyzed, with follow-up from 30 October 2004 to 30 October 2012. The log-rank test was used to compare survival between the two groups. A Cox proportional hazards model was used to determine hazard ratios to identify prognostic factors. The study included 521 patients in the TCM + cART group followed up for 3548 person-years and 375 patients in the cART group followed up for 2523 person-years. Mortality rates were 3.2/100 person-years and 4.2/100 person-years in the TCM + cART and cART groups, respectively. The difference in survival was significant. After adjusting for explanatory variables, the mortality rate of AIDS patients in the cART group was 1.7 times higher than in the TCM + cART group. Male sex, older age, little education, and lower CD4 cell count were risk factors for mortality. TCM intervention in addition to cART could increase survival of AIDS patients.

## 1. Introduction

There were 35 million people living with HIV globally in 2013, and only 12.9 million had access to combination antiretroviral therapy (cART), as reported by Joint United Nations Programme on HIV/AIDS (UNAIDS) [1]. It is well established that cART reduces mortality and improves the quality of life of HIV patients [2]. However, there are large variations in treatment outcomes because of differences in adherence to cART [3, 4], which can be poor because of adverse effects of cART, such as nausea, diarrhea, fatigue, lipodystrophy, and skin disorders [5, 6]. These adverse effects have required researchers to investigate new drugs or complementary treatments. Traditional Chinese medicine (TCM) has been used for thousands of years for many diseases. It has been used to treat HIV patients for many years, with amelioration of symptoms and a reduction in the adverse effects of cART [7].

In central China in the early mid-1990s, plasma donation was promoted among poor, rural farmers as an easy way to supplement their low income, and this resulted in large numbers contracting HIV infection [8]. In response to this situation, HIV-infected farmers were identified in 2004 in this area, and the Chinese government established the National Free Antiretroviral Treatment Program (NFATP) there [9]. Meanwhile, the Chinese National Administration of Traditional Chinese Medicine started a National TCM HIV Treatment Trial Program (NTCMTP), and central China was one of the locations chosen for its introduction [10].

By the end of 2012, the NTCMTP had been in place for 8 years. During the treatment program, HIV patients reported a major improvement in their clinical symptoms, and there was a significant reduction in opportunistic infections. However, the long-term clinical benefits of NTCMTP are unclear. Therefore, the purpose of our analysis was to document the long-term effect of NTCMTP in terms of survival of AIDS patients and its determinants through a retrospective cohort study.

## 2. Materials and Methods

### 2.1. Procedure of NTCMTP

When the State Administration of Traditional Chinese Medicine started the NTCMTP in central China, the areas enrolled were those in which an official TCM service was already available. The clinicians contacted HIV patients in the selected areas and explained the nature of the program. If the patients consented to attend the program voluntarily, further education was given on treatment adherence. The participants were given the fixed preparation,* yi ai kang* capsules (contenting ginseng,* huangqi*,* chaobaishu*, Tuckahoe, Chinese angelica,* chuanxiong*,* baishao*, and* Scutellariae*), and ingested five capsules three times daily. Additional TCM treatment was given according to the individual patient's symptoms. At the enrolment visit, the baseline characteristics of the participants were noted on a case report form (CRF), including epidemiological information, laboratory measurements (i.e., CD4+ T-cell count, whole blood cell count, urine analysis, liver and renal function, and HIV viral load), details of therapy, clinical symptoms, and vital status. Clinicians reviewed the participants every month, gave free* yi ai kang* capsules, and completed the details in the CRF. When a participant did not return for his or her medicine, clinicians actively investigated whether the participant had died or was lost to follow-up. Participants could also attend for free investigation and treatment of illnesses occurring between scheduled appointments.

### 2.2. Study Population

All participants in this study lived in the area of Henan Province, located in central of China, selected for the NTCMTP in October 2004. The inclusion criteria for this retrospective cohort study were HIV infection identified by western blot before 30 October 2004; age older than 18 years and younger than 65 years on 30 October 2004; being still alive on 30 October 2004; having taken cART between 30 October 2003 and 30 October 2004; and continuing taking cART at follow-up. Individuals enrolled in the NTCMTP were included as the TCM + cART group and those not enrolled were included as the cART group. Participants were followed up from October 2004 to October 2012.

### 2.3. Data Collection and Variables

Data were collected from standard medical record registers adopted by the office of the NTCMTP or Center for Disease Control and Prevention (CDC), Henan. There were three registers used in this study: the first included all HIV-positive patients enrolled in the NTCMTP; the second included all confirmed HIV-positive patients; and the third included all patients who started cART and had follow-up information. The first database belonged to the Administration of Traditional Chinese Medicine, and the latter two databases belonged to the CDC; thus there was no overlap between TCM + cART and cART therapy groups. The criteria for cART administration were in accordance with the NFATP.

Most information in this study such as demographics, method of HIV diagnosis, route of infection, data of death, and cause of death was collected from the second register. The first register provided data on the date of commencement of TCM therapy, follow-up data, and reasons for loss to follow-up. The third register provided data on starting and stopping cART. Data on CD4+ cell count in 2004 were collected from all three registers.

### 2.4. Data Analysis

The endpoint was the date of death, without consideration of the cause of death. The patient was censored at the time of loss to follow-up or at the end of follow-up in October 2012. Life table analysis was used to compute the cumulative survival rates. Kaplan-Meier and log-rank tests were used to compare survival curves in the two groups. The Cox proportional hazard model was used to identify factors associated with outcome. The adjusted hazard ratio (AHR) for death and 95% confidence interval (CI) were determined. All statistical analyses were performed by SPSS version 19.0 (IBM Corp., Armonk, NY, USA). Two-sided *P* values <0.05 were considered statistically significant.

### 2.5. Ethical Considerations

This study was approved by the institutional review board of the First Hospital affiliated to Henan University of Traditional Chinese Medicine. Individual informed consent was not required because this analysis used currently existing data collected during the course of routine treatment, and the data were anonymized for inclusion in the analysis.

## 3. Results

### 3.1. Patients

**Figure 1:**
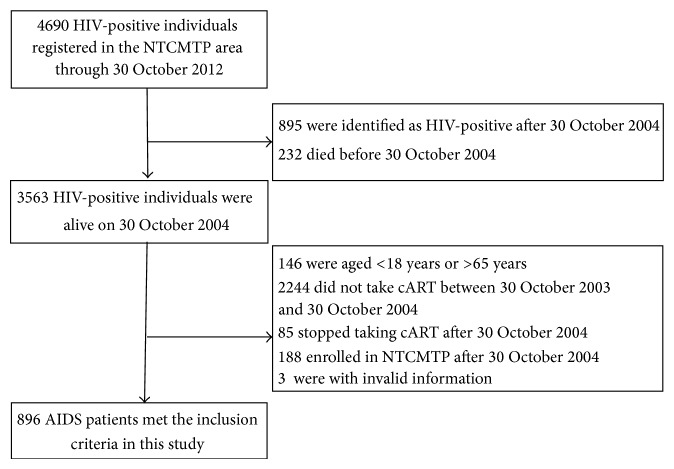
Profile of the study cohort. TCM, traditional Chinese medicine; cART, combination antiretroviral therapy; NTCMTP, National Traditional Chinese Medicine HIV Treatment Trial Program; AIDS, acquired immune deficiency syndrome.

By the end of 30 October 2012, 4,690 HIV-positive individuals were registered in the second register, 3563 of whom were identified as HIV-positive as of October 2004, and 896 met the inclusion criteria for this study (Figure 1). There were 521 patients in the TCM + cART group and 375 in the cART group. The mean age of the TCM + cART group was 41.8 ± 7.9 years, 519 (99.6%) were farmers, and 513 (98.5%) were infected with HIV through plasma donation, with 421 (80.8%) diagnosed between October 2003 and October 2004. The mean age of the cART group was 41.7 ± 7.8 years, 372 (99.2%) were farmers, and 368 (98.1%) were infected with HIV through plasma donation, with 277 (73.9%) diagnosed between October 2003 and October 2004. The baseline characteristics of the patients at initiation of the study are shown in Table 1.

### 3.2. Mortality Rate and Cumulative Survival

**Figure 2:**
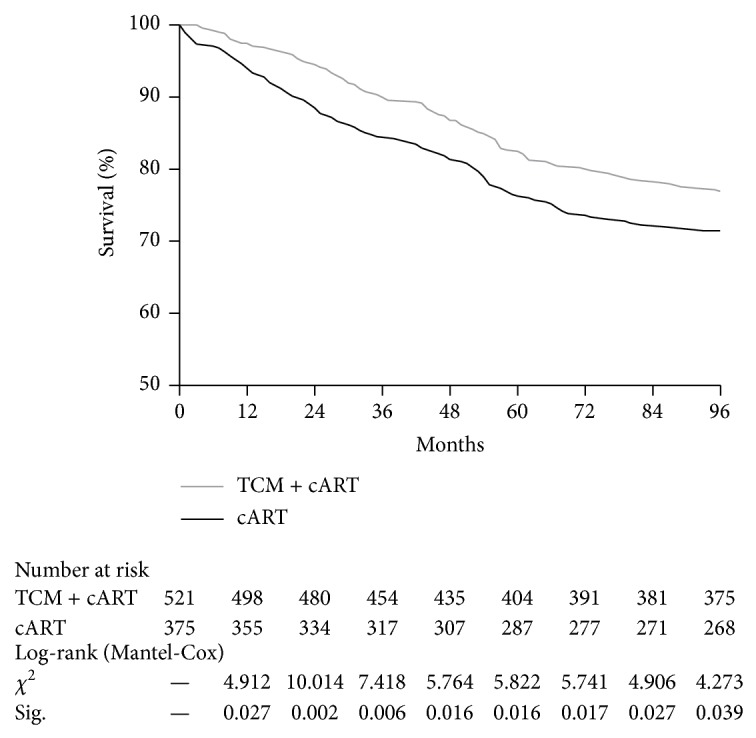
Comparison of cumulative survival between the two groups of AIDS patients.

In the TCM + cART group, patients were followed up for 3548 person-years, 114 (22.9%) died, and 32 (6.1%) were lost to follow-up. The mortality rate was 3.2/100 person-years; 97.5% of patients were alive after 1 year, 90.4% after 3 years, 82.7% after 5 years, and 77.2% after 8 years. The reasons for loss to follow-up were as follows: 11 left home for work; 5 dropped out because of adverse drug effects; and no reason was given for the other 16. In the cART group, patients were followed up for 2523 person-years, and 107 (28.5%) died. The mortality rate was 4.2/100 person-years; 94.7% of patients were alive after 1 year, 84.5% after 3 years, 76.5% after 5 years, and 71.5% after 8 years. The difference in cumulative survival between the two groups was statistically significant (Figure 2).

### 3.3. Factors Associated with AIDS-Related Death

Table 2 shows the results of Cox proportional hazard model analysis. The HR for mortality in the cART group was 1.3 times that of the TCM + cART group by single factor analysis [HR: 1.32, 95% confidence interval (CI): (1.01, 1.72)]. After adjusting for the baseline factors in multivariate analysis, the AHR was 1.7 times higher in the cART group compared with the TCM + cART group [AHR: 1.65, 95% CI (1.24, 2.18)].

The AHR was approximately 2.2-fold higher in male than female patients [AHR: 2.23, 95% CI (1.69, 2.96)]. Patients aged 50 years or older had higher AHR than patients aged 40 years or younger [AHR: 2.52, 95% CI (1.77, 3.57)]. Patients educated for more than 6 years had lower AHR than patients educated for less than 6 years [AHR: 0.71, 95% CI (0.52, 0.97)]. With an increase in CD4+ T-cell count, there was an increase in survival time; the AHR was lower in patients with CD4+ T-cell counts of 200–350 cell/mm^3^ [AHR: 0.51, 95% CI (0.37, 0.71)], 350–500 cell/mm^3^ [AHR: 0.36, 95% CI (0.23, 0.59)], and >500 cell/mm^3^ [AHR: 0.18, 95% CI (0.09, 0.34)] compared with patients with <200 cell/mm^3^.

## 4. Discussion

This retrospective cohort study showed that the mortality rate of HIV patients in the TCM + cART group was significantly lower than that of patients in the cART group. The HIV-related mortality rate varies across different countries because of differences in patient compliance, quality of treatment services, and characteristics of the patients [11–13]. The mortality rate of adults with AIDS was reported to be 5/100 person-years after 6 months of receiving free cART treatment in China [10]. A study from Henan—the same area as in the current study—found a mortality rate of 5.1/100 person-years after cART [14], which was slightly higher than in the cART group of this study. This may be because some patients had already been taking cART for more than 3 months at the start of this study, and other studies have reported that mortality was highest in the first 3 months after cART initiation [15]. Another study reported that the mortality rate was 3.6/100 person-years after TCM without consideration of cART in the same area as this study [16]. This was higher than in patients in the TCM + cART group in the current study.

The objective of most survival analyses of HIV patients is to estimate the time to death, and different methodological approaches have been reported. Some studies take the date of diagnosis of AIDS as the start for the cohort [17, 18], while others may include HIV-infected individuals in their cohort regardless of clinical status at baseline [19]. The aim of this study was to explore the effect of TCM on mortality of HIV patients treated with cART, so HIV patients at different clinical stages were included. The study showed that the mortality rate of HIV patients only taking cART was 1.7 times higher than HIV patients who took TCM and cART, after adjusting for other explanatory variables.

As a type of complementary and alternative medicine, the characteristic of TCM is to treat disease based on syndrome differentiation. According to the syndrome of HIV patients in Henan, TCM experts developed the* yi ai kang* capsules.* Yi ai kang* was the drug of choice in the NTCMTP in Henan, and the clinical effect showed that it could help to relieve clinical symptoms, decrease HIV viral load, increase the CD4 lymphocyte count, and decrease mortality [20, 21]. This study also confirmed the long-term efficacy of TCM.

Mortality was associated with other factors in this study. Male HIV patients had a higher mortality risk and this was consistent with many studies across the world and in China [14, 22–24]. Men are more likely to smoke and consume alcohol [25, 26], which are risk factors for mortality. Men also seek treatment later and have lower compliance, which may also contribute to higher mortality in male than female HIV patients [23, 24]. The mortality rate of HIV patients older than 50 was 2.5 times that of HIV patients aged less than 40, possibly related to increases in both AIDS-related and unrelated illnesses with age [27, 28]. The baseline CD4 count was significantly inversely associated with mortality in this study, which was consistent with previous studies [11, 13, 16, 29].

This study included a large sample size and had a long-term follow-up. Almost all of the patients lived on a farm and were infected with HIV through plasma donation, so this was a simple and stable cohort, with little loss to follow-up. However, a major limitation of this study was that it was based on secondary data that were not collected for research purposes. Thus, important predictors of mortality, such as nutritional status, economic status, drug adherence, and drug resistance, were not collected. The baseline CD4 cell count information was not complete. The missing data could result in information basis. As in all observational cohort studies, unmeasured differences may exist among the patients under study.

## 5. Conclusion

Although this study is limited by its retrospective nature, it provides evidence that TCM intervention in addition to cART could increase survival of AIDS patients. As an effective AIDS therapy, TCM merits further research.

## Figures and Tables

**Table 1:** Patient characteristics at baseline, *n* (%).

Variables	All	TCM + cART	cART	Statistic value	*P* value
Total sample	896	521	375		
Gender					
Male	406 (45.3)	254 (48.8)	152 (40.5)	5.944	0.015
Female	490 (54.7)	267 (51.2)	223 (59.5)		
Age (years)					
<41	433 (48.3)	246 (47.2)	187 (49.9)	0.683	0.711
41–50	338 (37.7)	202 (38.8)	136 (36.3)		
>50	125 (14.0)	73 (14.0)	52 (13.9)		
Marital status					
Married	696 (77.7)	410 (78.7)	286 (76.3)	0.741	0.389
Single/widow(er)	200 (22.3)	286 (76.3)	89 (23.7)		
Education					
<6 years	600 (67.0)	349 (67.0)	251 (66.9)	0.001	0.987
>6 years	296 (33.0)	172 (33.0)	124 (33.1)		
CD4+ T-cell count (cell/mm^3^)					
<200	233 (26.0)	141 (27.1)	92 (24.5)	90.197	0.001
200–350	240 (26.8)	160 (30.7)	80 (21.3)		
351–500	134 (15.0)	94 (18.0)	40 (10.7)		
>500	115 (12.8)	79 (15.2)	36 (9.6)		
Unknown	174 (19.4)	47 (9.2)	127 (33.9)		

TCM, traditional Chinese medicine; cART, combination antiretroviral treatment.

**Table 2:** Hazard ratios for AIDS-related death for baseline variables.

Categories	Cases	Deaths	Unadjusted		Adjusted	
			HR (95% CI)	*P* value	AHR (95% CI)	*P* value
Group						
TCM + cART	521	115	1		1	
cART	375	107	1.32 (1.01, 1.72)	0.04	1.65 (1.24, 2.18)	0.001
Gender						
Female	490	92	1		1	
Male	406	130	1.87 (1.43, 2.44)	0.001	2.23 (1.69, 2.96)	0.001
Age (years)						
<41	433	92	1		1	
41–50	338	74	1.02 (0.75, 1.38)	0.919	1.13 (0.83, 1.55)	0.439
>50	126	56	2.43 (1.74, 3.39)	0.001	2.52 (1.77, 3.57)	0.001
Marital status						
Married	696	153	1		1	
Single/widow(er)	200	47	0.91 (0.66, 1.26)	0.574	0.75 (0.54, 1.04)	0.088
Education level						
<6 years	600	160	1		1	
>6 years	296	62	0.76 (0.57, 1.02)	0.066	0.71 (0.52, 0.97)	0.032
CD4+ T-cell count (cell/mm^3^)						
<200	233	95	1		1	
200–350	240	59	0.51 (0.37, 0.70)	0.001	0.51 (0.37, 0.71)	0.001
351–500	134	21	0.31 (0.19, 0.50)	0.001	0.36 (0.23, 0.59)	0.001
>500	115	10	0.17 (0.09, 0.32)	0.001	0.18 (0.09, 0.34)	0.001
Unknown	174	37	0.42 (0.28, 0.61)	0.001	0.36 (0.24, 0.54)	0.001

HR, hazard ratio; AHR, adjusted hazard ratio; CI, confidence interval.
